# Loss of mucin 2 and MHC II molecules causes rare resistance to murine RV infection

**DOI:** 10.1128/jvi.01507-24

**Published:** 2024-12-27

**Authors:** Carolyn Bomidi, Faith M. Sawyer, Noah Shroyer, Margaret Conner, Mary K. Estes, Sarah E. Blutt

**Affiliations:** 1 Department of Molecular Virology and Microbiology, Baylor College of Medicine 189531 , Houston, Texas, USA; 2 Section of Gastroenterology and Hepatology, Department of Medicine, Baylor College of Medicine 686421 , Houston, Texas, USA; 3 Department of Medicine, Baylor College of Medicine 171841 , Houston, Texas, USA; University of Michigan Medical School, Ann Arbor, Michigan, USA

**Keywords:** RV, resistance, pathogenesis, receptor, tropism, Atoh1, Muc2, secretory cell, MHC II

## Abstract

Enteric pathogen rotavirus (RV) primarily infects mature enterocytes at the tips of the intestinal villi; however, the role of secretory Paneth and goblet cells in RV pathogenesis remains unappreciated. Atoh1 knockout mice (Atoh1cKO) were used to conditionally delete Paneth, goblet, and enteroendocrine cells in the epithelium to investigate the role of secretory cells in RV infection. Unexpectedly, the number of infected enterocytes and the amount of RV shedding in the stool were greatly decreased following secretory cell deletion. Resistance to RV infection persisted for 7 days after virus inoculation, and Atoh1 knockout mice co-housed with infected wild-type mice were uninfected, based on lack of shedding virus, despite the highly infectious nature of RV. This response was directly proportional to the extent of secretory cell deletion, with infection predominantly occurring in areas containing intact secretory cells. RV infection of *Muc2* knockout mice recapitulated the secretory cell deletion phenotype, indicating that goblet cell loss is responsible for attenuated infection. Transcriptome analysis of Atoh1cKO intestine via single-cell RNA sequencing revealed downregulation of MHC II molecules specifically in tip enterocytes, and MHC II^-/-^ mice were likewise resistant to RV infection. These data suggest a previously unknown role for both MUC2 and MHC II expression in susceptibility to RV infection.

**IMPORTANCE:**

Rotavirus (RV) is a highly contagious pathogen that primarily infects mature intestinal enterocytes. Murine rotavirus readily infects infant and adult mice, enabling evaluation of RV infection and immunity. We report that mice lacking secretory cells are one of the few genetically modified mouse lines not susceptible to murine rotavirus. Further investigation revealed loss of mucin 2 (MUC2) expression or major histocompatibility complex II (MCH II) expression recapitulated this rare resistance to rotavirus infection, suggesting a previously unrecognized link between secretory cell products and major histocompatibility complex II expression. Furthermore, these mouse models provide a platform to investigate rotavirus pathogenesis.

## INTRODUCTION

Rotavirus (RV) is the most common cause of gastroenteritis in children younger than 2 years old, resulting in varying severity of dehydrating diarrhea, vomiting, and fever (1–4). RV also infects adults who experience mild to severe disease symptoms (5–7). In the small intestine, RV exhibits limited tissue tropism, infecting mature enterocytes, enteroendocrine cells, and tuft cells primarily in the upper villi as well as the follicle-associated epithelium (2, 3, 8–12). Infection by RV is thought to be a receptor-mediated process although a cellular receptor for RV has not been identified (2, 13). RV is a non-enveloped virus containing an 11 segment, double-stranded RNA genome and it belongs to the *Reoviridae* family (2, 4). The genome encodes both structural and non-structural proteins and is enclosed within a triple-layered protein capsid (4). The outer capsid protein VP7 and the spike protein VP4 are thought to mediate binding and entry (2, 13) although host factors also affect cellular attachment and uptake. Sialoglycans, integrins, heat shock proteins, mucins, and histo-blood group antigens have all been implicated in aspects of RV infection and pathogenesis (2, 13).

The small intestinal villus epithelium is populated by two broad categories of cells: absorptive and secretory cells. Absorptive enterocytes represent approximately 90% of the epithelium and are responsible for uptake of luminal nutrients (14). Secretory cells include goblet cells that produce mucus, Paneth cells that produce antimicrobial peptides and growth factors, enteroendocrine cells (EECs) that produce hormones, and immune-modulatory tuft cells (14). Intestinal stem cells reside in the crypts of Lieberkühn where they divide to provide daughter cells that terminally differentiate into absorptive and secretory cells following a specific program of transcription factor and growth factor gradient cues (14, 15). The transcription factor Atoh1 (also known as Math1) must be expressed to commit cells to the secretory pathway and remains expressed at basal levels in secretory cells during maturation (16). To produce enterocytes, Notch signaling induces Hes1, a transcriptional repressor, which in turn inhibits Atoh1 expression (17, 18). Secretory Paneth and goblet cells share a common progenitor, characterized by GFI1 expression; GFI1 represses the EEC fate gene *Ngn3* to encourage Paneth and goblet cell specification (19). Paneth cells are then specified by increased Wingless-related integration site (WNT) signaling to induce *Sox9* gene expression (20). In contrast, goblet cells are specified following reduced WNT signaling and activation of transcription factors SPDEF and KLF4 (16, 21, 22). In the absence of GFI1, EECs differentiate due to NGN3 expression, and SOX4 expression is instrumental to drive homeostatic numbers of both tuft cells and EECs (23–26). Tuft cells are specified following Pou2f3 gene expression although both *Atoh1*-dependent and *Atoh1*-independent tuft cell specification pathways have been proposed (23, 27–30).

Mice provide an excellent model system in which to explore how the intestinal epithelium interacts with, and responds to, pathogens. Adult mice are readily susceptible to infection with several murine strains of RV, and infection is easily monitored via detection of viral proteins in fecal pellets. Mice excrete viral proteins as early as 24 hours following oral inoculation, exhibit peak amounts of fecal virus loads between 4 and 5 days, and resolve infection by 10 to 11 days (31–34). Although RV primarily infects mature enterocytes, recent identification of RV in non-enterocyte populations (8–11) suggests that secretory cells may play a more significant role in RV pathogenesis than previously appreciated. To investigate the role of secretory cells in RV pathogenesis, we inoculated mice that lack intestinal secretory cells (Atoh1cKO) with murine RV and examined susceptibility to infection. Atoh1cKO mice demonstrated unexpected resistance to murine RV infection with little to no viral excretion following inoculation. Loss of mucin 2 (MUC2) and major histocompatibility complex II (MHC II) expression partially recapitulated this resistance phenotype, suggesting potential mechanisms through which secretory cells regulate RV susceptibility.

## RESULTS

### Atoh1cKO mice are resistant to murine RV infection

**Fig 1:**
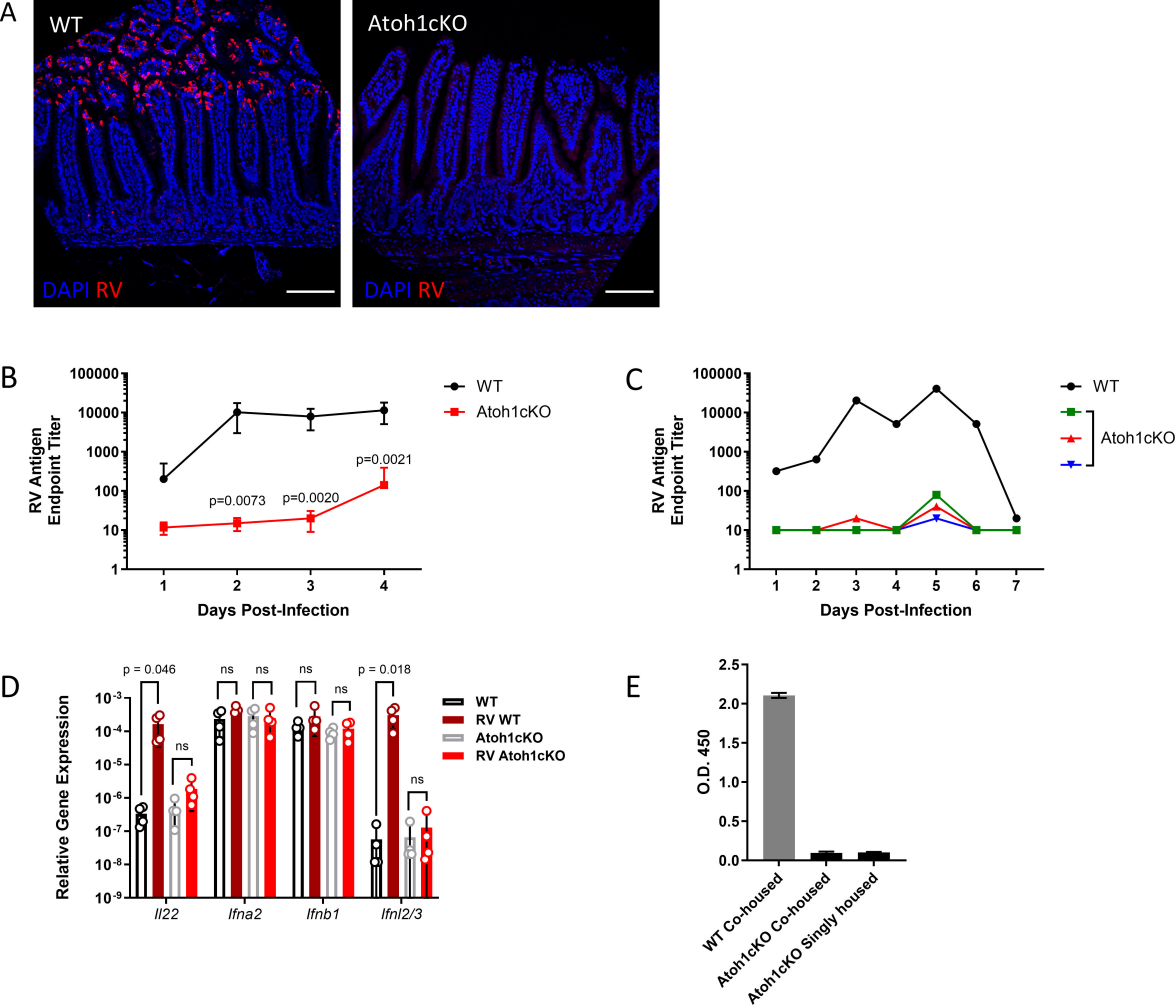
Mice lacking intestinal secretory cells are resistant to murine rotavirus infection. (**A**) Immunofluorescence of Atoh1cKO (right) or littermate control (WT, left) intestine at 4 days post-infection (dpi) with rotavirus EC_wt_ (RV). Scale bar: 100 µm. Red: RV, blue: nuclei. (**B**) Fecal ELISA monitoring RV shedding in WT or Atoh1cKO stool for 4 dpi. Unpaired *t*-test results are indicated by *P* value; all other comparisons are not significant (*P* value > 0.05). *n* = 4–6 mice per group; symbols indicate mean ± SD. (**C**) Fecal ELISA monitoring RV shedding in representative WT or Atoh1cKO stool for 7 dpi. Each line indicates shedding from a single mouse. (**D**) RNA was isolated from the jejunum of WT and Atoh1cKO mice, and qRT-PCR was used to quantify various immune response transcripts at 4 dpi. *n* = 4 mice per group; bars show mean ± SD. Unpaired *t*-test results are indicated by brackets. ns: not significant (*P* value > 0.05). (**E**) A WT mouse was infected with RV and co-housed with uninfected Atoh1cKO mice and WT littermate controls. Two additional Atoh1cKO mice were infected and singly housed for comparison. RV shedding was assessed via fecal ELISA 4 days after exposure to RV or the infected WT mouse.

Conditional deletion of transcription factor *Atoh1* in the intestinal epithelium of mice causes loss of secretory cells (Paneth, goblet, and EECs) as documented by histology, reduced gene expression of secretory cell markers, loss of immunostaining of markers of secretory cells, and potential increase in tuft cells (15, 27, 29, 35, 36) (Fig. S1). Although *Atoh1*-deleted crypt-villus units are morphologically normal, proliferating cells fill the intestinal crypts and the epithelium demonstrates a deficient response to injury (35). To investigate the effect of secretory cell loss on RV infection, we conditionally deleted Atoh1 from intestinal epithelial cells in *Vil1^Cre/ERT2^*^+^; *Atoh1^fl/fl^* (Atoh1cKO) mice and inoculated with murine RV strain EC_wt_. In agreement with previous reports, littermate control animals with intact secretory cells (WT) demonstrated RV infection in mature enterocytes at the tips of jejunal intestinal villi, and viral proteins were readily detectable in fecal pellets (Fig. 1A through C). In contrast, Atoh1cKO mice had very few infected cells and shed much lower or undetectable levels of RV (Fig. 1A through C). Consistent with very low levels of infection, transcripts of immune mediators *Il22* and *Ifnl2/3*, previously shown to be upregulated following robust RV infection, were not significantly increased in the infected Atoh1cKO intestine as compared to uninfected cKO littermates (Fig. 1D) (37, 38). Other immune transcripts, *Ifna2a* and *Ifnb1*, which are not associated with robust RV infection, were not different between Atoh1cKO and WT littermates (37, 38) (Fig. 1D). Co-housing mice excreting RV routinely results in horizontal transmission of infection to uninfected cage mates due to the highly transmissible nature of murine RV infection (39). However, Atoh1cKO mice co-housed with an infected WT littermate were not susceptible to horizontal transmission; the Atoh1cKO mice remained uninfected by RV (Fig. 1E). Taken together, these data indicate that intestinal expression of Atoh1 is strictly required for productive RV infection.

### Resistance to RV infection is dose responsive and localized in Atoh1KO mice

**Fig 2:**
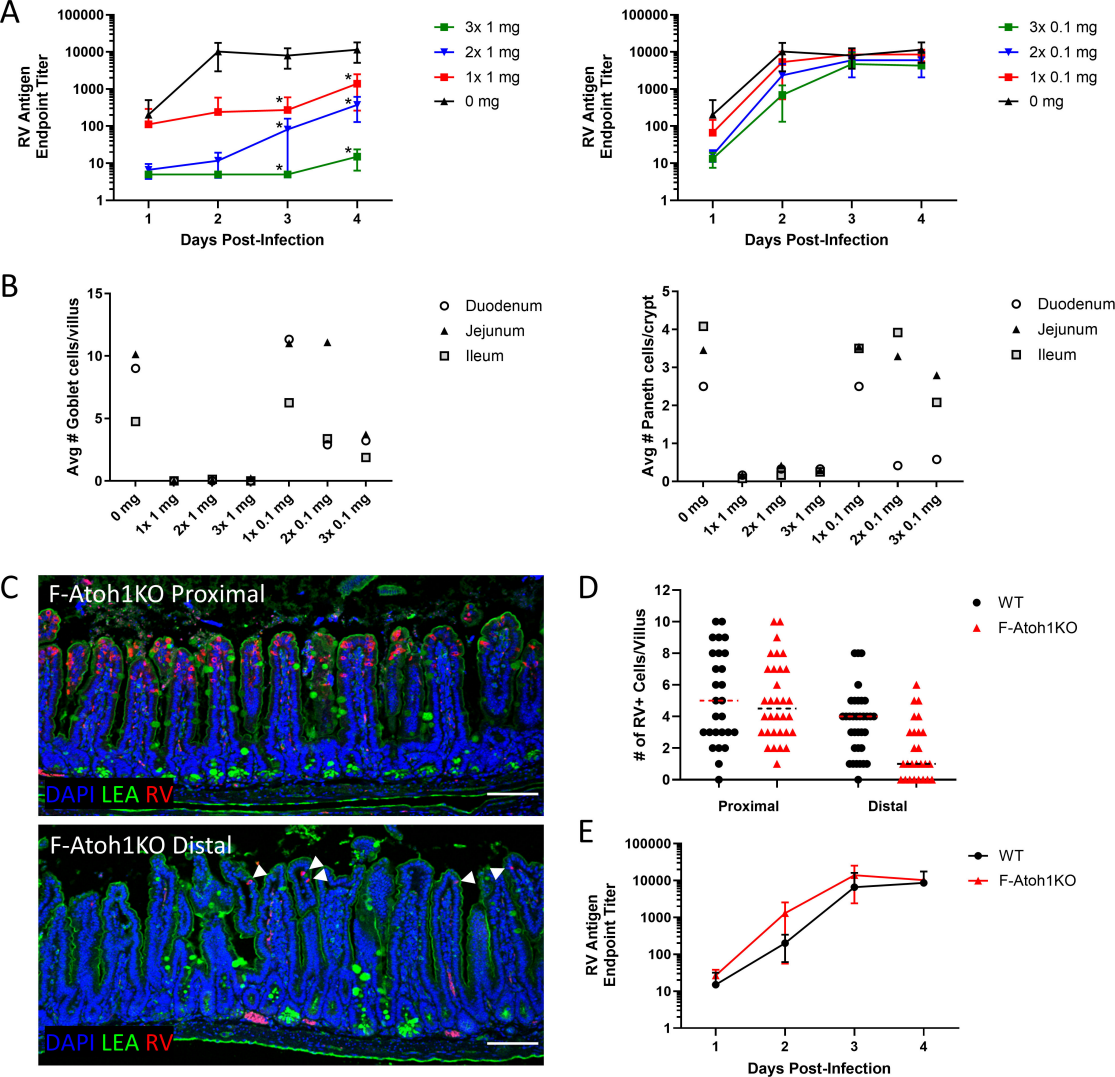
Resistance to murine RV is titratable and localized. (**A**) Fecal shedding of *Vil1^Cre/ERT2+^*; *Atoh1fl/fl* mice treated with one to three doses of either 1 mg (left) or 0.1 mg (right) tamoxifen and infected with RV. Significant unpaired *t*-test results between no tamoxifen (0 mg) and one to three doses of tamoxifen are indicated by asterisks; all other comparisons are not significant (*P* value > 0.05). *n* = 3–4 mice per condition; symbols indicate mean ± SD. (**B**) The average number of goblet (left) and Paneth (right) cells was assessed via PAS stain at 4 dpi. Goblet and Paneth cells from at least 20 well-oriented crypts or villi were counted per segment per animal. *n* = 3 animals averaged per condition. (**C**) Immunofluorescence of region-specific Atoh1KO (F-Atoh1KO) intestine at 4 dpi. FITC-conjugated LEA lectin (green) marks Paneth and goblet cells as well as surface glycans. Red: RV, blue: nuclei. White arrowheads indicate infected cells. Red signal below the crypts indicates nonspecific staining of red blood cells. Scale bar = 100 µm. (**D**) Number of infected cells per villus in the proximal or distal intestine of F-Atoh1KO and littermate controls (WT) from (**C**). >25 well-oriented villi from infected animals were selected for quantification. Dotted line indicates median. (**E**) Fecal shedding of *Fabp1*^*Cre*^; *Atoh1*^*fl/fl*^ (F-Atoh1KO) mice and littermate controls (WT) infected with RV. *n* = 3–4 animals per condition; symbols indicate mean ± SD.

To determine the extent of *Atoh1* deletion required to resist RV infection, we examined whether partial deletion of secretory cells restored RV infectivity. Secretory cell numbers were controlled by titrating of the amount of tamoxifen each animal received. Partial depletion of secretory cells with low doses of tamoxifen restored infection in correlation with the number of remaining secretory cells (Fig. 2A and B). To assess the extent of the Atoh1KO effect, we infected mice that lacked secretory cells in the distal ileum and colon due to mosaic Cre expression controlled by the transcriptional regulatory elements of *Fabp1* (*Fabp1^Cre^*; *Atoh1^fl/fl^*, F-Atoh1KO) (40). Tissue sections from F-Atoh1KO mice were stained with *Lycopersicum esculentum* lectin conjugated with fluorescein (FITC-LEA) to label both Paneth cells and goblet cells, and an anti-RV antibody to identify infected cells. Infected enterocytes were located only in crypt-villus units with intact goblet and Paneth cells or adjacent to villi with intact secretory cells, within two crypt-villus units (Fig. 2C), indicating a relationship between the presence of secretory cells and the susceptibility of enterocytes to infection. Despite fewer infected cells in the distal intestine of F-Atoh1KO mice (Fig. 2D), RV excretion in F-Atoh1KO mice was similar to levels in *Cre*^-/-^ littermate controls (WT) (Fig. 2E), consistent with the results from the tamoxifen titration experiments (Fig. 2A and B) in which restoration of some secretory cells restored WT excretion.

### *Muc2* deletion, but not Paneth cell depletion, recapitulates Atoh1cKO resistance

Atoh1 deletion results in global loss of secretory cells, which encompasses goblet cells, Paneth cells, and EECs (35, 36) (Fig. S1A through C). To determine whether a particular secretory cell was essential for RV infectivity, we examined the response to RV infection in mice lacking various secretory cell-associated genes. As Paneth cells make many antimicrobial factors that can influence pathogenesis of intestinal pathogens, we utilized a variety of mouse models to determine whether the lack of Paneth cells or Paneth cell-derived factors influenced susceptibility to RV infection. *Spdef^-/-^* and conditionally deleted Gfi1 (*Vil1^CreERT2/+^*; *Gfi1^flox/flox^*, Gfi1cKO) mice lack expression of transcription factors that promote maturation of Paneth cells and goblet cells. Specifically, Gfi1cKO mice lack Paneth cells and specify fewer goblet cells (19), and *Spdef^-/-^*mice specify modestly decreased numbers of goblet cells and significantly reduced numbers of Paneth cells (41). RV infection of these mice revealed no differences in RV shedding in fecal pellets or in the numbers of RV-positive enterocytes at the villus tips when compared to WT littermates (Fig. S2 and S3). Likewise, mice that conditionally lacked the ability to produce Paneth cell effectors, such as *Defa2* (Defa2-DTR) or lysozyme (*Lyz*^*CreERT2/+*^-DTA mice, Lyz-DTA), also had little to no alterations in RV excretion or in the number of infected enterocytes as compared to littermate controls (Fig. S2 and S3). These results indicate that a lack of Paneth cells was not responsible for the reduced susceptibility to RV infection observed in Atoh1cKO mice.

**Fig 3:**
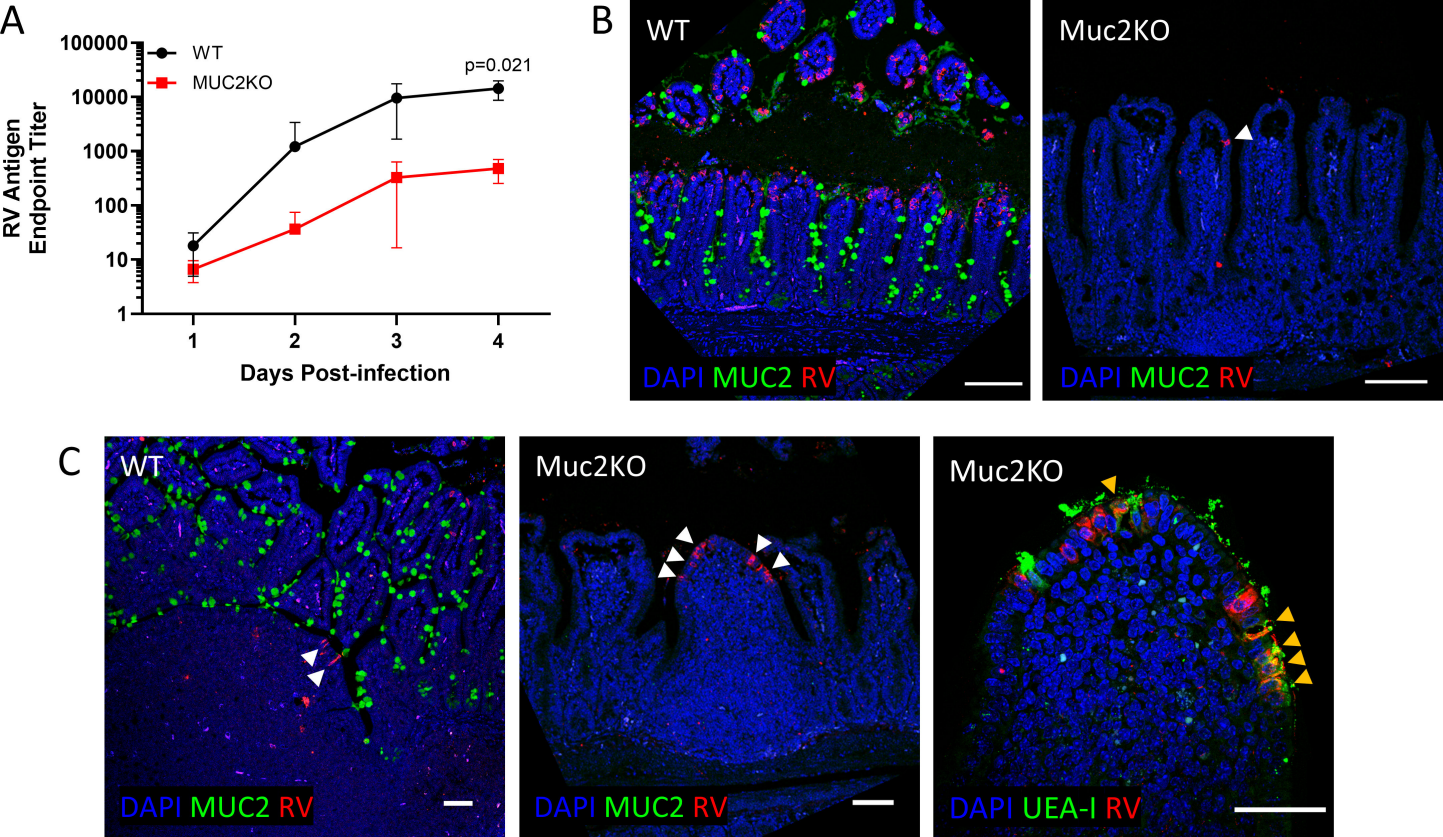
Tip enterocytes are resistant to RV infection in Muc2KO mice, but the follicle-associated epithelium is susceptible. (**A**) Fecal RV ELISA from Muc2KO mice and littermate controls (WT). *n* = 3–4 animals per line; symbols indicate mean ± SD. Unpaired *t*-test results are indicated by *P* value; all other comparisons are not significant (*P* value > 0.05). (B) Immunofluorescent staining for MUC2 (green), RV (red), and nuclei (blue) at 4 days post-infection (dpi). White arrowhead indicates rare infected enterocyte. Scale bar = 100 µm. (C) Infected WT or Muc2KO intestine stained with MUC2 antibody (left) or FITC-conjugated lectin Ulex europaeus agglutinin (UEA-I, right) and anti-RV at 4 dpi. White arrowheads indicate infected cells in the follicle-associated epithelium (left). Yellow arrowheads indicate cells double-positive for UEA-I (green) and RV (red) (right). Scale bar = 50 µm.

Mucins, produced by goblet cells, have been implicated in RV pathogenesis (42, 43). To investigate whether MUC2, the predominant mucin produced by intestinal goblet cells, influenced susceptibility to RV infection, we infected *Muc2*^-/-^ mice (Muc2KO) and WT littermate controls with RV and examined goblet cell morphology and infection kinetics. As expected, tissue sections from Muc2KO mice displayed small, depleted goblet cells following periodic acid-Schiff (PAS) stain (Fig. S4) (44, 45). Muc2KO mice infected with RV shed significantly less RV antigen than their WT littermates (Fig. 3A), and infected enterocytes were rarely detected (Fig. 3B), similar to results in Atoh1cKO mice (Fig. 1A through C). In contrast to Atoh1cKO mice, which demonstrated very few infected cells, RV antigen was primarily detected in the follicle-associated epithelium of MucKO mice, specif`ically in UEA-I positive M cells (Fig. 3C). Based on these findings, the loss of the goblet cell product MUC2 results in a decreased susceptibility of tip enterocytes to RV infection.

### MHC II expression on tip enterocytes depends on secretory cells and is required for RV infection

**Fig 4:**
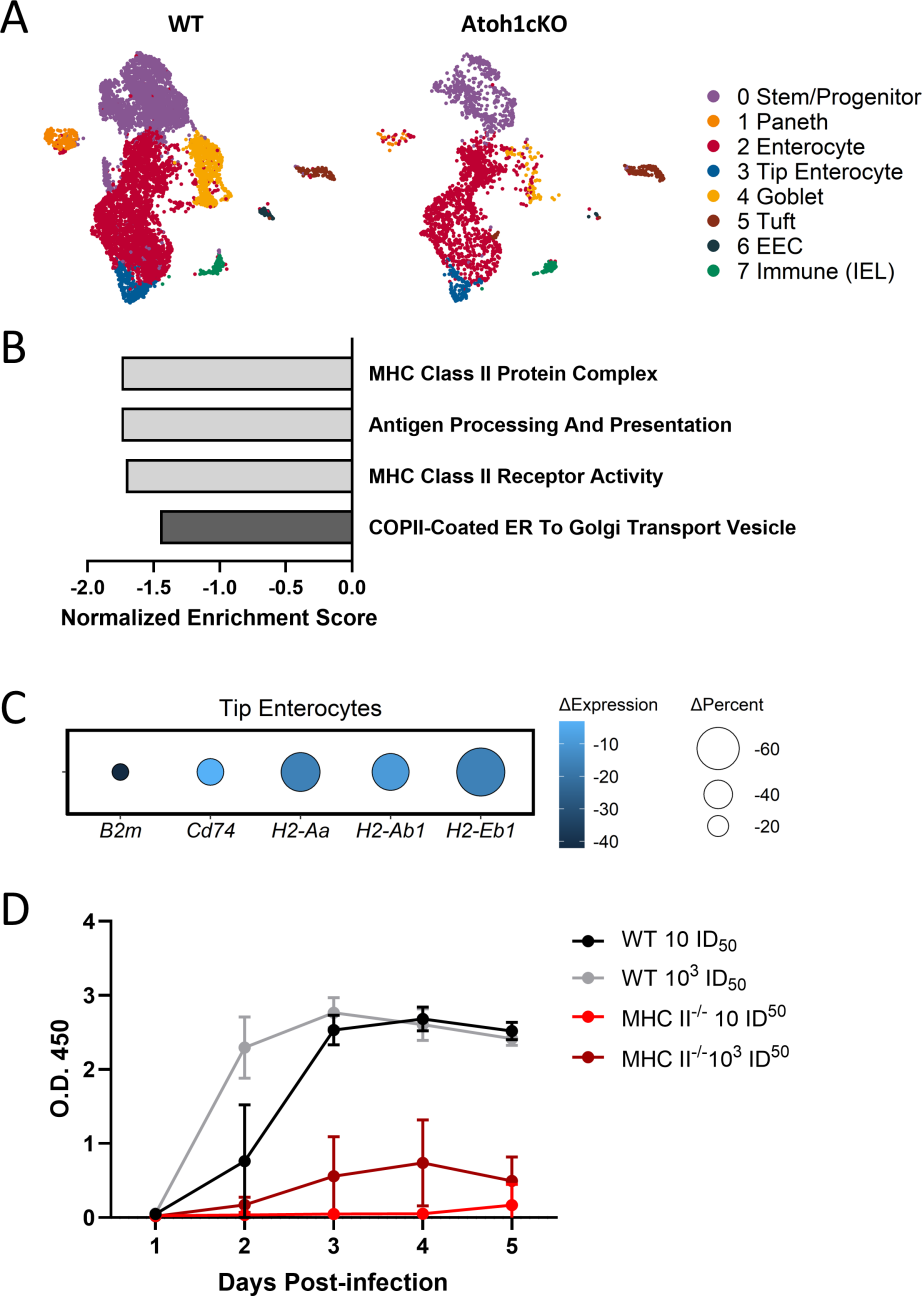
Enterocyte MHC II expression is decreased in Atoh1cKO mice and MHC II-/- mice are resistant to RV infection (A) Tip enterocytes (blue) were identified in a previously published Atoh1cKO scRNAseq dataset (29). IEL: Intraepithelial lymphocytes. (B) Select de-enriched Gene Ontology (GO) pathways in Atoh1cKO tip enterocytes versus WT tip enterocytes. p value < 0.05. (C) Differentially expressed genes associated with the GO pathways in (B). Adjusted p value < 0.05. ∆Percent indicates change in percent of cells expressing the gene (Atoh1cKO versus WT), ∆Expression indicates change in mean expression level. (D) MHC II-/- mice were infected with low (10 ID50) or high (1E3 ID50) dose of rotavirus (RV). Stool was collected daily to quantify viral load via RV ELISA. n=4-6 mice per group; symbols indicate mean ± SD.

We hypothesized that villus tip enterocytes, the main site of RV replication, were altered following loss of secretory cells, thereby making them less susceptible to RV infection. However, Atoh1cKO and Muc2KO tip enterocytes did not display altered morphology despite the absence or alteration of secretory cells (Fig. S1 and S4). Furthermore, surface staining with fluorescein isothiocyanate (FITC)-conjugated lectins revealed that Atoh1cKO tip enterocytes retain surface glycans implicated in RV attachment and entry (46) (Fig. S5A). To determine whether there were transcriptional changes in Atoh1cKO tip enterocytes that might explain the alterations in RV susceptibility, we analyzed a published Atoh1cKO single-cell RNA sequencing (scRNAseq) data set (29), as an equivalent Muc2KO data set is not available. Tip enterocytes, the main site of RV replication, were identified by expression of known mature enterocyte regional genes including *Ada, Apoa1,* and *Apoa4* and by the absence of mid-villus marker gene *Slc2a2* (47, 48) (Fig. 4A; Fig. S5B and S5C). Gene set enrichment analysis revealed that Atoh1cKO tip enterocytes have decreased expression of genes associated with MHC II pathways and vesicle transport, such as *H2-Aa* and *H2-Ab1* (Fig. 4B and C). To determine if a lack of MHC II expression has a relationship to RV susceptibility, we infected mice lacking five MHC II complex genes (MHC II^-/-^) (49) with a low and high dose of RV. Viral excretion was monitored in fecal pellets during the course of infection. MHC II^-/-^ mice exhibited a phenotype similar to that of Atoh1cKO and Muc2KO mice, with severely reduced RV excretion compared to WT C57BL/6 controls (Fig. 4D). These data strongly suggest a role for both MUC2 and MHC II expression in susceptibility to RV infection.

## DISCUSSION

The development of intestinal secretory cells depends on expression of *Atoh1*, yet the overall morphology of the intestinal epithelium, including crypt-villus regional organization, remains intact following loss of *Atoh1* expression (35, 36). Here, we report that Atoh1cKO mice, which lack secretory cell populations, are resistant to infection with murine RV, despite no previous known role for secretory cells in RV replication. In over 40 years of RV research, only one other murine model has shown to be resistant to murine RV infection. In that model, *Candidatus Arthromitus*, commonly referred to as segmented filamentous bacteria (SFB), was able to prevent and cure RV infection (31, 50, 51). However, SFB 16S qPCR of Atoh1cKO stool demonstrated similar or lower levels of SFB as compared to WT mice (Fig. S6A), and SFB-specific fluorescent *in situ* hybridization did not detect elevated SFB levels in Atoh1cKO intestine (Fig. S6B), indicating the mechanism of resistance to RV is independent of SFB level (31, 52). Given that the exact mechanism by which SFB confer resistance to RV remains poorly understood, there may be overlap in the mechanisms of action between SFB and loss of secretory cells or MHCII. Nevertheless, the data presented suggest that Atoh1cKO mice are resistant to RV infection via an SFB-independent mechanism.

Our finding that Muc2KO mice are resistant to RV infection (Fig. 3) indicates that goblet cell deficiency is likely responsible for the Atoh1cKO phenotype. This result was unexpected given that goblet cells have been shown to play a role in defense against RV infection. Several groups have reported that pre-incubation of RV with mucins causes reduced infection *in vitro* (42, 53). Because RV can directly bind to mucins, it has been postulated that mucus serves as a decoy receptor that aids in clearing RV infection (42, 43, 54). Consistent with this idea, bacteria able to degrade mucin glycans enhance RV infection *in vitro* (31, 42). The finding that *Muc2* deletion leads to resistance to RV infection indicates that a complete lack of mucus is inhibitory, rather than beneficial, suggesting that the role of mucins in RV infection is more nuanced than previously suspected. The altered infection pattern in Muc2KO mice, shifting from enterocytes to primarily M cells (Fig. 3C), suggests that RV requires some amount of mucin glycans to enter enterocytes, whereas M cell luminal sampling, and thus likely RV transport (55), remains intact.

The finding that mature Atoh1cKO enterocytes have significantly reduced MHC II expression and that MHC II^-/-^ mice were also resistant to RV infection was also unexpected (Fig. 4C and D). MHC II proteins are constitutively expressed on human intestinal enterocytes located in the upper villus, the main site of RV infection (56–59), suggesting that MHC II may be a molecular determinant of tropism for RV infection. MHC II is also expressed on the basolateral surface of enterocytes, which is interesting given that some strains of RV preferentially infect enterocytes at the basolateral surface (60–64). These results hint at an important role for MHC II molecules in early stages of RV infection, possibly in vesicle transport necessary for establishing sites of RV replication (Fig. 4B). Although this observation is highly preliminary and warrants further study *in vitro*, it is complementary to reports that MHC II is an essential entry mediator for bat influenza A virus defining its tropism; mice lacking MHC II are resistant to bat influenza infection, whereas WT mice are not (65). Together, these data present new avenues for studying RV tropism and early requirements for entry that may reveal a definitive receptor for RV.

The result that the goblet cell product MUC2 is required for high levels of RV infection highlights an unexpected connection between secretory cells and MHC II expression. The exact link between secretory cell specification via ATOH1*,* including MUC2 expression, and MHC II levels remains unclear; however, a possible mechanism may be found in how goblet cells regulate the intestinal microbiota. Loss of MUC2 alters the composition of the microbiota, which ultimately results in inflammation or cancer (45, 66). Likewise, MHC II expression has been tied to the microbiota, as the intestinal epithelium in germ-free mice is devoid of MHC II expression prior to colonization with exogenous bacteria (67–69). Thus, we hypothesize that deficient expression of MUC2 causes a shift in the microbiota’s composition that results in less MHC II expression on enterocytes, which is ultimately responsible for a corresponding decrease in RV infection. This effect is likely related to common bacterial composition, as evidenced by the complementary findings from the KO lines reported here and the scRNAseq analysis of Atoh1cKO mice raised at a different institution (29). Further study is needed to understand how secretory cell products influence MHC II expression and how bacterial composition affects susceptibility to RV.

Our findings indicate a functional role for MUC2 and MHC II molecules in RV pathogenesis, suggesting that manipulating secretory cell products may be a therapeutic strategy for curbing RV infection. Beyond RV pathogenesis, the indication that secretory cells can influence the expression profile and function of mature enterocytes is an exciting new discovery with wider implications for our understanding of intestinal homeostasis and disease.

## MATERIALS AND METHODS

### Mouse lines and treatment

*Vil1^CreERT2/+^*; *Atoh1^flox/flox^* and *Vil1^CreERT2/+^*; *Gfi1^flox/flox^* mice were generated by crossing previously described Atoh1^f/f^ mice (35) or Gfi1^f/f^ mice (19) with *Vil1^CreERT2/+^* transgenic mice (70) purchased from The Jackson Laboratory (strain 020282). *FABP1^Cre^*; *Atoh1^flox/flox^* mice with mosaic expression of the rat FABP1^Cre^ transgene (35, 40) and *Spdef^-/-^* (22, 41) mice have been previously described. *Muc2^-/-^* mice were generated on a C57BL/6 background with CRISPR/Cas-mediated genome editing performed by the Genetically Engineered Rodent Models Core at Baylor College of Medicine as previously described (71, 72). Briefly, a small insertion was added in the start codon of *Muc2* causing a frameshift and truncation of the gene. Knockout was confirmed with genotyping PCR, Sanger sequencing, and tissue staining. MHC II^-/-^ mice purchased from The Jackson Laboratory (strain 003374) were bred in-house and compared to C57BL/6 mice, also purchased from The Jackson Laboratory (strain 101045) raised in-house. Lyz^CreERT2/+^-DTA mice (73) were kindly provided by Nan Gao. PC-DTR mice (74), a gift from Steve McElroy, were treated with one intraperitoneal (i.p.) dose of 10 ng/gbw diphtheria toxin (Millipore Sigma) 3 days prior to infection. CreERT2 recombinase activity was induced via i.p. injection of 1 mg of tamoxifen (Millipore Sigma) dissolved in ethanol and diluted in corn oil (10% ethanol final concentration) every other day, for a total of three doses, beginning 1 week prior to infection. *CreERT2*^*-/-*^ and *DTR*^*-/-*^ littermates were treated with tamoxifen or diphtheria, respectively, as WT controls. For RV infection, mice aged 8–18 weeks were infected via oral gavage of murine RV strain EC_WT_ (1 × 10^3^ infectious dose 50) or comparable volume of phosphate-buffered saline (PBS) homogenate unless otherwise indicated (75). RV shedding was quantified via stool enzyme-linked immunosorbent assay (ELISA) as previously described (75), and the reciprocal of the highest dilution that produced a value higher than the cutoff (OD_450_ 0.1) was reported as the RV antigen endpoint titer. For the co-housing experiment, a *CreERT2*^*-/-*^ mouse was infected with RV as above and housed with uninfected Atoh1cKO mice and littermate controls. Stool was collected from all co-housed mice for fecal RV ELISA at 4 and 8 dpi to confirm successful infection of the WT mouse and monitor infection of the cage mates. The Baylor College of Medicine Institutional Animal Care and Use Committee approved all animal protocols.

### Immunofluorescence and FISH

Murine small intestinal tissue was fixed, embedded, and stained as previously described (9). The antibodies used in this study are listed in Table S1. SFB localization was examined with a 16S FISH probe (IDT; GCTGCCTCCCGTAGGAGT) and SFB-specific FISH probe (IDT; GGGTACTTATTGCGTTTGCGACGGCAC) (52). Briefly, probes were diluted in hybridization buffer (1 M Tris HCl, NaCl2, 10% SDS), added to deparaffinized slides, and incubated sequentially for 1 hour in a hybridization oven at 51°C. Slides were washed in pre-warmed wash buffer (1 M Tris HCl, NaCl2) and counter stained with 4′,6-diamidino-2-phenylindole (DAPI; Invitrogen). SFB-enriched murine stool, used as a positive control, was provided by Andrew Gewirtz.

### qRT-PCR

Tissue RNA was isolated with a Direct-zol RNA Miniprep Kit (Zymo Research) with DNase I digestion according to the manufacturer’s protocol. Transcripts were quantified with the One-Step RT-qPCR ToughMix with ROX (Quanta Biosciences) on a StepOnePlus PCR System (Applied Biosystems). The TaqMan primers and probes (Thermo Fisher) are listed in Table S1. Transcript levels were normalized to *Gapdh* using the 2^-∆CT^ method (76).

### SFB 16S qPCR

Equal-volume fecal samples were collected at various timepoints during the course of the experiment and were bead beaten for 1 minute in 360 µL of buffer ATL (Qiagen), then incubated with 40 µL of Proteinase K (Qiagen) for 1 hour at 55°C. Nucleic acids were isolated from the homogenized samples with the DNeasy Blood and Tissue Kit (Qiagen) according to the manufacturer’s protocol. 16S quantification was performed with SYBR Green Supermix (Bio-Rad Laboratories) on the StepOnePlus PCR System (Applied Biosystems) with the SFB-specific 736F and 844R primer set (77).

### Single-cell RNA sequencing analysis

Data from a previously published scRNAseq on Atoh1cKO ileum (29) were downloaded from Gene Expression Omnibus (GEO). All WT and Atoh1KO1, Atoh1KO2_reseq, and Atoh1KO3 samples were combined into a single Seurat object and processed as previously described (9). Briefly, cells were filtered for less than 25% mitochondrial content, and total RNA content, percent mitochondria, and sample identity were regressed out during sctransform normalization. Villus tip enterocytes were identified at high-resolution clustering with previously published markers (47, 48), and Gene Set Enrichment Analysis was performed on differentially expressed genes ranked by log2FC as previously described (9).

## Data Availability

Previously published sequencing data from scRNA-seq (29) are available on NCBI. The GEO accession number for the scRNA-seq data re-analyzed in this paper is GSE145827.

## SUPPLEMENTAL MATERIAL

Supplemental material jvi.01507-24-s0001.pdf Figures S1 to S6 and Table S1.

ASM does not own the copyrights to Supplemental Material that may be linked to, or accessed through, an article. The authors have granted ASM a non-exclusive, world-wide license to publish the Supplemental Material files. Please contact the corresponding author directly for reuse.
