# Supplementary material for: Loss of mucin 2 and MHC II molecules causes rare resistance to murine RV infection
Source: J Virol. 2024 Dec 27;99(2):e01507-24. doi: 10.1128/jvi.01507-24 (PMC11852729; doi:10.1128/jvi.01507-24)
Supplement: Supplemental material — Figures S1 to S6 and Table S1. [file jvi.01507-24-s0001.pdf]

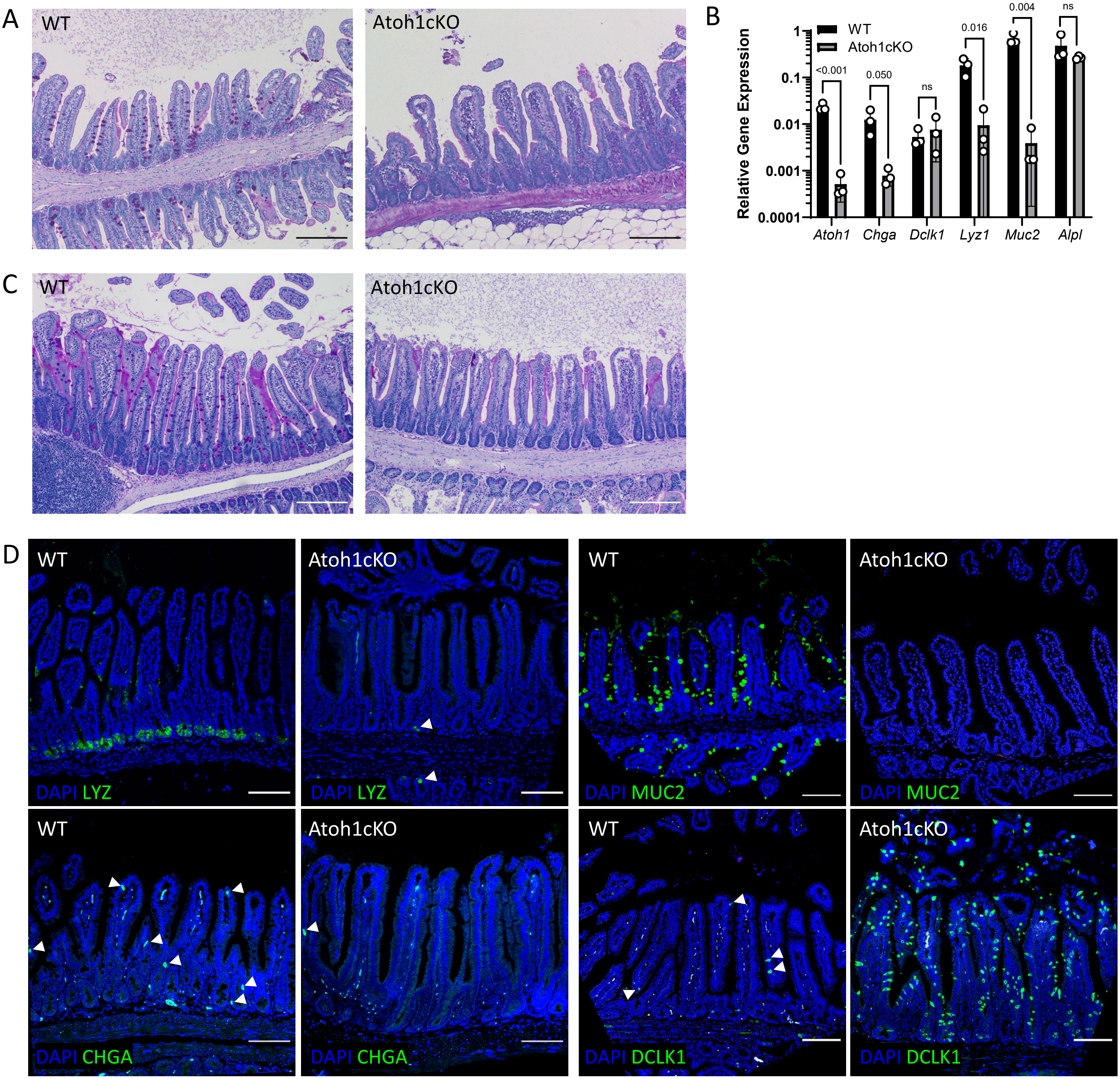

Figure S1. Conditional Atoh1 knockout results in loss of Paneth cells, goblet cells, enteroendocrine cells, and an increase in tuft cells.

(A) Representative PAS stain of littermate control (WT, left) or Atoh1cKO (right) intestine at 0 days post-infection (dpi). Scale bar = 100 µm.

(B) Expression levels of WT and Atoh1cKO secretory and absorptive markers relative to *Gapdh* prior to infection (0 dpi). n=3 mice per group; bars show mean ± SD. Unpaired t-test results are indicated by brackets and p value. Ns: not significant, p value > 0.05.

(C) Representative PAS stain of WT (left) or Atoh1cKO (right) intestine at 4 dpi. Scale bar = 100 µm.

(D) Representative immunofluorescence of secretory markers of WT or Atoh1cKO mice at 4 dpi. White arrow heads indicate rare positive cells. Scale bar = 100 µm.

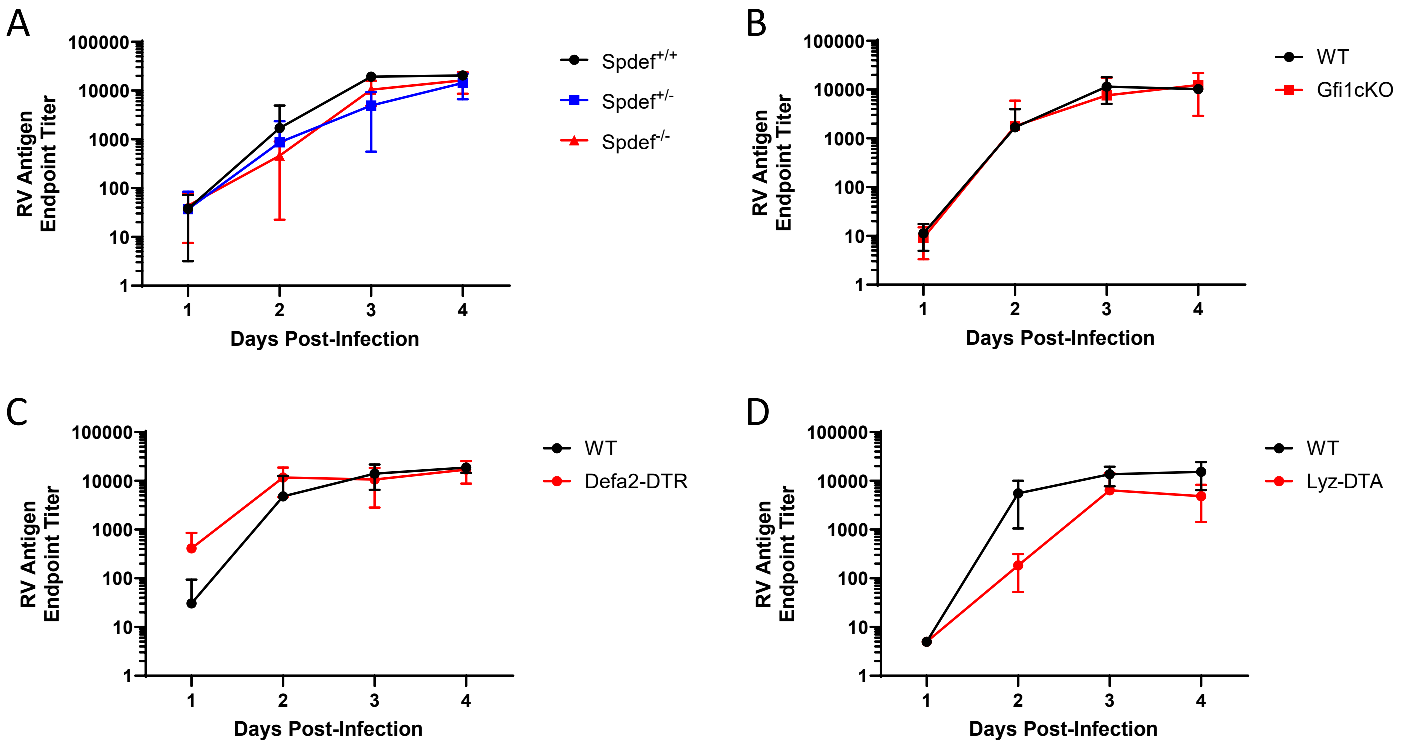

Figure S2. Paneth cell depletion does not reproduce Atoh1cKO resistance to rotavirus (RV).
Fecal RV ELISA from:

(A) $Spdef^{-/-}$ mice and littermate controls
(B) Gfi1cKO mice and littermate controls
(C) Defa2-DTR mice and littermate controls
(D) Lyz-DTA mice and littermate controls

n=3-4 mice per group; symbols indicate mean ± SD. All unpaired t-test comparisons are not significant (p value > 0.05).

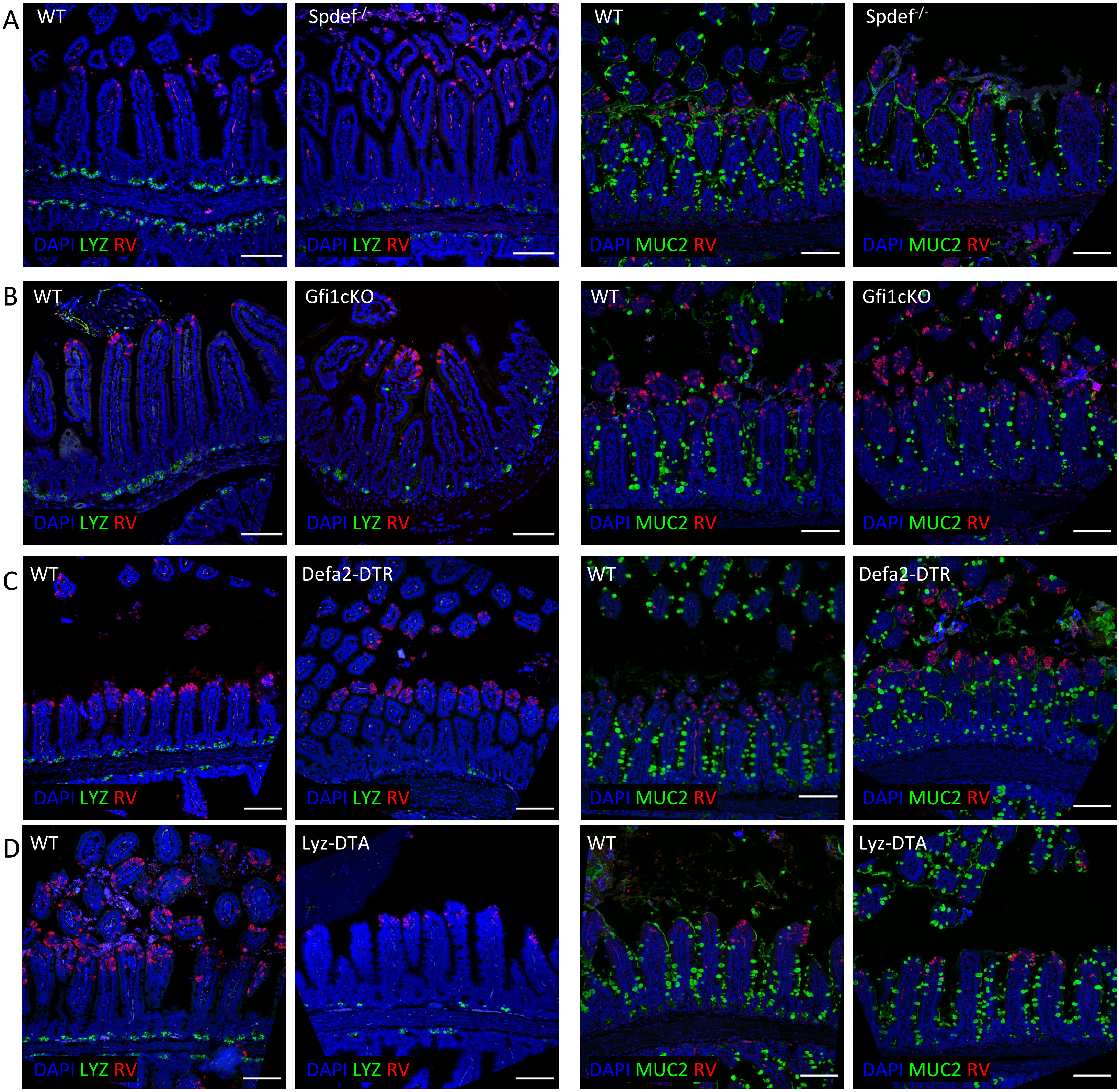

Figure S3. Paneth cell depletion does not reproduce Atoh1cKO resistance to RV.
Representative immunofluorescence images (red: RV, green: LYZ (left) or MUC2 (right), blue: nuclei) from various KO lines at 4 days post-infection. Scale bar = 100 µm.

(A) Spdef$^{-/-}$ mice and littermate controls
(B) Gfi1cKO mice and littermate controls
(C) Defa2-DTR mice and littermate controls
(D) Lyz-DTA mice and littermate controls

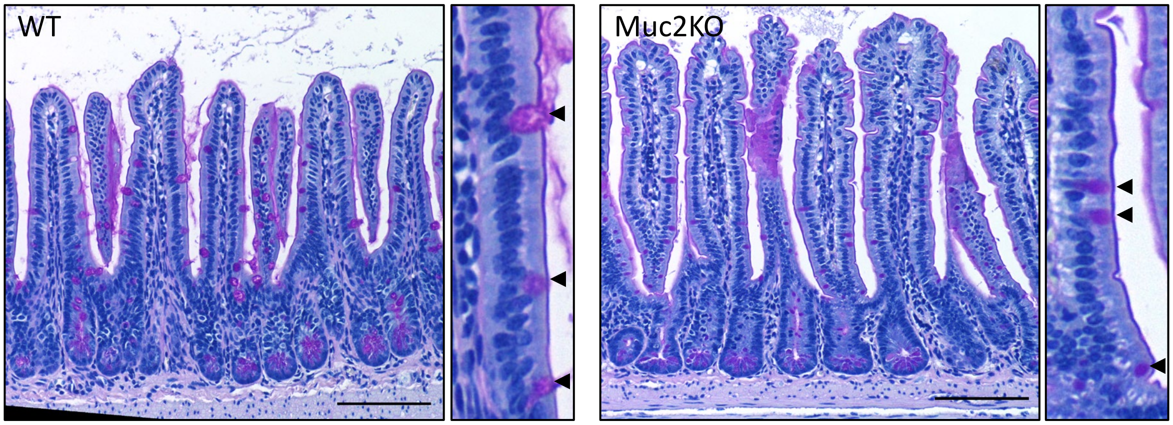

Figure S4: PAS stain of littermate (WT) and Muc2KO mice 4 days post-infection reveals intact villi with altered goblet cell morphology. Black arrowheads on enlarged images indicate goblet cells. Scale bar = 100 µm.

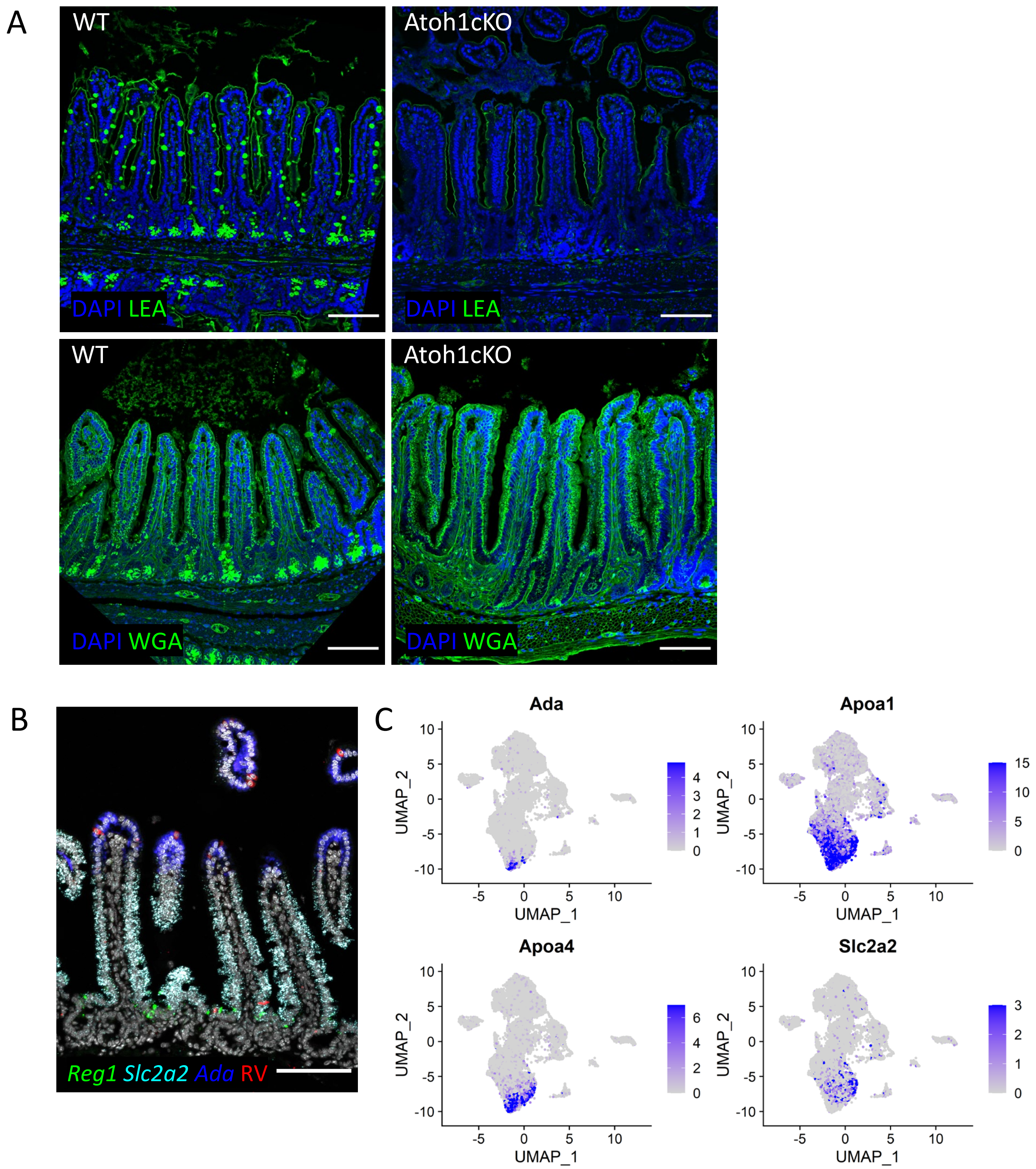

Figure S5. Atoh1cKO tip enterocytes retain glycan expression and RV infects enterocytes expressing *Ada.*

(A) FITC-conjugated lectin staining (top: Lycopersicon esculentum (LEA, in green), bottom: wheat germ agglutinin (WGA, in green) of littermate (WT) or Atoh1cKO intestine infected with rotavirus (RV) at 4 days post-infection (dpi). Scale bar: 100 µm.

(B) RNAscope® staining for villus-tip enterocyte gene *Ada* (blue), mid-villus gene *Slc2a2* (cyan), and lower-villus gene *Reg1* (green) and immunofluorescent staining for RV (red) in a C57BL/6 mouse at 4 dpi. Scale bar: 100 µm

(C) Distribution of tip enterocyte gene expression (*Ada*, *Apoa1*, and *Apoa4*) as well as mid-villus marker *Slc2a2* in the combined WT and Atoh1cKO epithelium scRNAseq dataset UMAP.

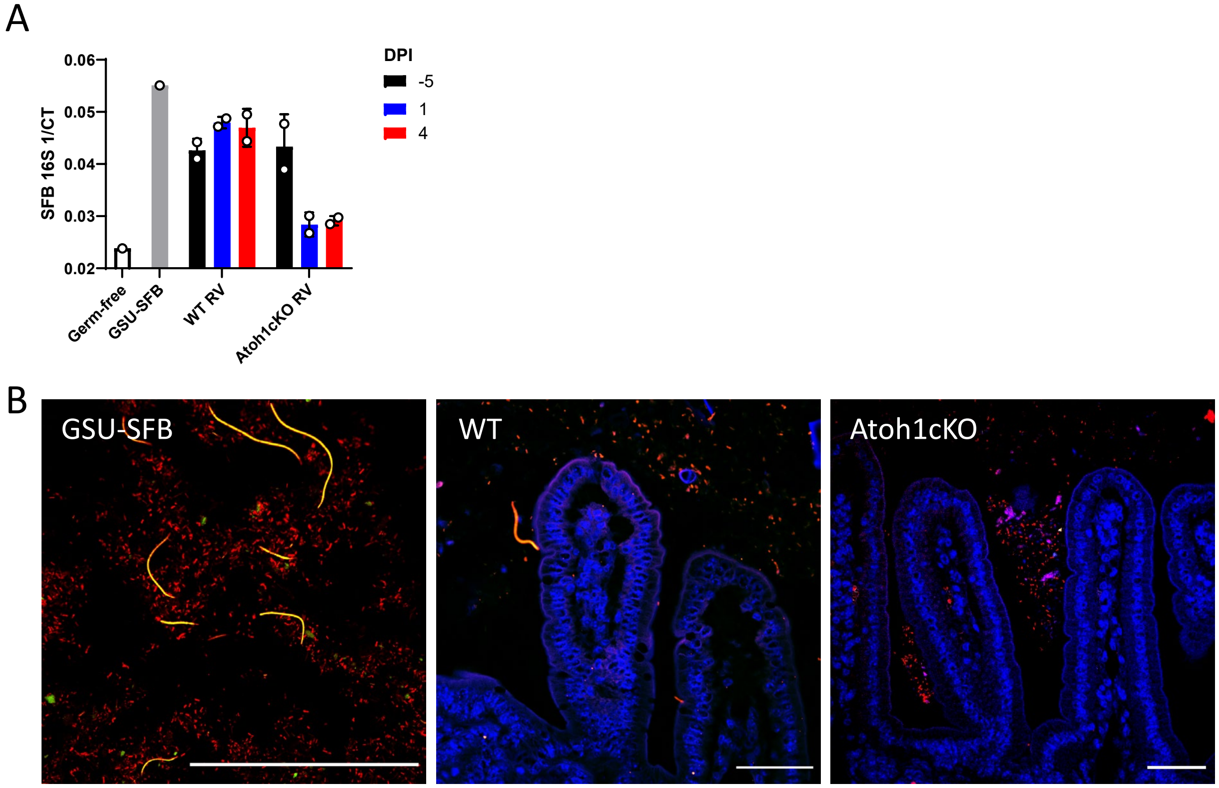

Figure S6. Atoh1cKO resistance to RV infection is not due to high SFB load

(A) DNA was isolated from stool samples from infected littermate (WT) and Atoh1cKO mice at different days post-infection (dpi) and SFB level was quantified by 16S qPCR. Germ-free and SFB-enriched (GSU-SFB) fecal samples were included for comparison. SFB was undetected in the germ-free sample; CT set to the maximum cycle number. Bars represent mean ± SD.

(B) Fluorescent in situ hybridization of GSU-SFB fecal smear (left), WT (middle), and Atoh1cKO (right) intestines at 0 dpi. Red: pan-16S, green: SFB, blue: nuclei, yellow: merge. Scale bar: 50 µm.

Table S1 Reagents

Reagent	Source	Catalog No.	Note
**Antibodies**			
RV (GP511)	Laboratory-generated	-	1:400
LYZ1	Dako	A0099	1:1000
CHGA	Immunostar	20085	1:1000
DCLK1	Cell Signaling	62257	1:300
MUC2	Abcam	ab272692	1:1000
FITC-LEA	Vector Labs	FL-1171	5 µg/ml
FITC-WGA	Vector Labs	FL-1021	5 µg/ml
FITC-UEA-I	Vector Labs	FL-1061	5 µg/ml
**qRT-PCR probes**			
Gapdh	ThermoFisher	Mm99999915_g1	
Atoh1	ThermoFisher	Mm00476035_s1	
Chga	ThermoFisher	Mm00514341_m1	
Dclk1	ThermoFisher	Mm00444950_m1	
Lyz1	ThermoFisher	Mm00657323_m1	
Muc2	ThermoFisher	Mm01276696_m1	
Alpi	ThermoFisher	Mm01285814_g1	
Il22	ThermoFisher	Mm01226722_g1	
Infb1	ThermoFisher	Mm00439552_s1	
Ifna2	ThermoFisher	Mm00833961_s1	
Ifnl2/3	ThermoFisher	Mm04204158_gH	
**RNAScope® probes**			
Ada	ACD Bio	562501	
Slc2a2	ACD Bio	439891	
Reg1	ACD Bio	511571	
